# The NutriLight framework: a novel approach to evaluating sustainable and healthy diets

**DOI:** 10.1017/jns.2025.10015

**Published:** 2025-07-14

**Authors:** Tingyu Lu, Weiyu Chen, Xiaochun Huang, Manyi Zhai, Chunqiao Fu, Lin Xu

**Affiliations:** 1 School of Public Health, Sun Yat-sen University, Guangzhou, China; 2 Greater Bay Area Public Health Research Collaboration, Guangdong, China; 3 School of Public Health, The University of Hong Kong, Hong Kong, Hong Kong; 4 Institute of Applied Health Research, University of Birmingham, Birmingham, UK

**Keywords:** Dietary patterns, Environmental impact, Health and sustainability, Nutritional adequacy, NutriLight scoring system, Sustainable Plate, BMI, Body mass index, DASH, Dietary approaches to stop hypertension, HEI, Healthy eating index, AHEI, Alternative healthy eating index, UNEP, United Nations Environment Programme

## Abstract

The NutriLight system presents a novel dietary approach designed to enhance health communication, promote sustainable eating habits, and address limitations in existing dietary patterns. Using a traffic light scoring system, it simplifies dietary recommendations, making them more accessible and adaptable across diverse populations. Unlike rigid diets, NutriLight categorises foods into green, yellow, and red groups, encouraging balance rather than restriction. This flexibility allows for cultural adaptations, ensuring relevance in different dietary contexts while supporting planetary health. Additionally, NutriLight mitigates the risk of nutrient deficiencies by emphasising whole, minimally processed foods and reducing overconsumption of unhealthy options. While promising, its effectiveness depends on proper implementation, localised adaptation, and long-term evaluation to confirm its health benefits. By bridging the gap between nutritional science and practical application, NutriLight has the potential to serve as an effective tool in public health nutrition, fostering healthier and more sustainable dietary choices worldwide.

## Introduction

Dietary patterns represent the types, quantities, and frequencies of food and beverage consumption, tailored to address specific health and lifestyle objectives such as managing chronic diseases,^(1,2)^ weight control,^(3)^ or environmental sustainability.^(4)^ While over ten recognised dietary patterns, including the Mediterranean, Dietary approaches to stop hyptertension (DASH), and ketogenic diets, have emerged globally, their universal application is constrained by several challenges. Health communication barriers, such as complex guidelines^(5)^ and misinformation,^(6)^ complicate their adoption. Cultural diversity also limits global applicability, as many popular dietary frameworks are rooted in Western food traditions and fail to account for regional preferences or availability.^(7–9)^ Furthermore, concerns about long-term health impacts and nutritional adequacy in restrictive diets, like ketogenic^(10)^ or vegan patterns,^(11)^ highlight the necessity for dietary strategies that are both inclusive and scientifically robust. To overcome these limitations and enhance the integration of sustainable and health-promoting diets, we propose the concept of the Sustainable Plate.

The Sustainable Plate is a holistic and practical framework designed to promote healthy eating habits while addressing environmental sustainability. It combines the principles of plant-based diets^(12)^ and planetary health diets,^(13)^ offering an accessible model for facilitating health communication and encouraging positive dietary changes. Inspired by the Traffic Light System,^(14)^ the Sustainable Plate categorises foods into three groups: green, yellow, and red lights. Green light foods, such as whole grains, vegetables, fruits, soy and legumes, and nuts, are emphasised for their superior nutritional profiles and low environmental footprints. Yellow light foods, including refined grains, poultry, dairy foods, eggs, fish and seafood, plant oils and starchy vegetables, are recommended in moderation to balance dietary variety with sustainability. Red light foods, such as red meat, animal oils, and added sugars, are strictly limited due to their significant environmental and health costs.

Sustainable dietary practices require a significant shift toward increased consumption of plant-based foods, including vegetables, fruits, whole grains, legumes, nuts, and seeds, while reducing reliance on animal-based products, particularly red and processed meats.^(15,16)^ This transition is critical for simultaneously promoting human health and environmental sustainability, as plant-based diets are associated with lower greenhouse gas emissions and resource use. Additionally, dietary guidelines should integrate environmental sustainability to align public health goals with ecological priorities. Such guidelines must assess the environmental impact of different dietary patterns and provide clear recommendations for sustainable food choices. However, the lack of standardised methods for assessing and reporting dietary practices limits the comparability of research findings, reducing their applicability in developing effective dietary guidelines.^(17–19)^ Standardised frameworks are therefore essential to synthesise evidence and enable practical translation into public policies.

Unlike traditional dietary guidelines that rely heavily on information dissemination, the Sustainable Plate framework leverages behavioural insights to enhance real-world applicability. The NutriLight system simplifies dietary choices through an intuitive traffic-light approach, making it easier for individuals to adopt healthier eating patterns without requiring extensive nutritional knowledge. Additionally, by allowing regional adaptations, it increases relevance and feasibility across different populations, addressing limitations seen in the past public health dietary models. The Sustainable Plate not only promotes personal well-being but also advances collective efforts toward a sustainable food future, offering a pathway to harmonise public health and planetary health in dietary discourse.

## Theoretical framework

The Sustainable Plate is underpinned by three guiding principles: nutrient adequacy, environmental impact, and cultural acceptability, which collectively provide a holistic framework for promoting healthy and sustainable dietary patterns. These principles address the multifaceted challenges of ensuring dietary practices that are health-promoting, environmentally sound, and socially relevant, offering a comprehensive framework to align public health goals with ecological and cultural priorities.

### Nutrient adequacy

Nutrient adequacy emphasises the consumption of a variety of foods to meet essential dietary requirements and prevent nutrient deficiencies. Similar to the concept of dietary diversity outlined in the perspectives, the Sustainable Plate prioritises the inclusion of a wide range of nutrient-dense foods, particularly whole grains, vegetables, fruits, soy and legumes, and nuts. These foods not only enhance nutritional quality but also contribute to overall health outcomes, aligning with evidence that diverse diets promote nutritional adequacy and reduce the risk of chronic diseases. Additionally, by discouraging overconsumption of nutrient-poor or excess-calorie foods, the Sustainable Plate ensures balance and moderation, fostering dietary patterns that support both individual health and sustainability.

### Environmental impact

Environmental sustainability is a central tenet of the Sustainable Plate, which aims to reduce the ecological footprint of food systems. This aligns with research highlighting the need for dietary shifts towards plant-based patterns to mitigate environmental degradation caused by intensive livestock farming and resource-heavy food production.^(20)^ Drawing from multidimensional dietary frameworks, the Sustainable Plate recognises that the environmental costs of food choices must be evaluated alongside their nutritional benefits. For instance, the categorisation of foods into ‘green’, ‘yellow’, and ‘red’ groups reflects an integration of nutrient adequacy with environmental considerations, where green light foods are both nutritionally optimal and environmentally sustainable.

### Cultural acceptability

Cultural acceptability ensures that dietary patterns resonate with the diverse traditions, preferences, and social contexts of global populations.^(21,22)^ Recognising the importance of localised food practices, the Sustainable Plate emphasises tailoring dietary recommendations to regional and cultural contexts. The framework allows flexibility within its green, yellow, and red light categories to accommodate variations in food availability and cultural preferences, promoting inclusivity and reducing resistance to adoption. One key reason that past dietary models struggled with adherence was their lack of cultural adaptability. This flexibility ensures that the NutriLight system remains applicable in different populations while maintaining core health and sustainability principles.

Taste is central to food choices. Sustainable dietary shifts must prioritise flavour enhancement, culinary adaptation, and food reformulation to make plant-based options more appealing. Modifying food environments, through menu design, industry collaboration, and sensory-based nudges, can further support this transition.

However, access to green-light foods such as fruits and nuts can be limited by economic and geographic disparities. High costs, seasonal fluctuations, and supply chain constraints may restrict availability in certain regions. To address these challenges, the framework supports incorporating locally available nutrient-dense alternatives (e.g. legumes, seeds, or region-specific fruits) that maintain dietary balance while enhancing affordability. Furthermore, improving food distribution systems and implementing policy interventions, such as targeted subsidies for nutrient-rich foods, can help overcome these barriers, ensuring that the NutriLight system remains both culturally relevant and accessible.

## Components of a sustainable plate

The Sustainable Plate emphasises the role of dietary diversity within food groups to achieve both nutritional adequacy and sustainability (Table 1). The foundation of a Sustainable Plate is the prioritisation of plant-based foods such as vegetables, fruits, whole grains, soy and legumes, and nuts. These foods are central to dietary patterns like the Mediterranean and DASH diets, which are associated with improved health outcomes and lower environmental impacts compared to typical Western diets.^(23)^ Recommended intake amounts in Table 1 are based on the EAT-Lancet Commission reference diet, which provides evidence-based targets for achieving both human health and environmental sustainability.^(20)^ These guidelines align with global dietary recommendations and have been adapted within the NutriLight framework to promote balanced and sustainable food choices. However, individual dietary needs vary based on factors such as age, gender, physical activity level, and health status. The EAT-Lancet diet assumes an average intake of 2500 kcal/d, corresponding to a moderately active adult, but adjustments may be necessary for individuals with higher energy demands or specific nutritional needs, such as pregnancy or chronic disease management.^(20)^ The NutriLight framework allows for such modifications while maintaining its core principles of health and sustainability.

**Table 1.** The NutriLight recommendation for daily diets intake

Category	Food types	Recommended intake	Scoring
**Green Light Foods** (Recommended)	Whole grains, vegetables, fruits, soy and legumes, nuts	Whole grains: ≥ 232 g/d Vegetables: ≥ 300 g/d Fruits: ≥ 200 g/d Soy and legumes: ≥ 75 g/d Nuts: ≥ 50 g/d	≥ Recommended amount: **2 points** 0 < Intake < Recommended amount: **1 point** No intake: **0 points**
**Yellow Light Foods** (Moderate)	Refined grains, poultry, dairy foods, eggs, fish and seafood, plant oils, starchy vegetables	Refined grains: 100 g/d Poultry: 29 g/d Dairy: 250 g/d Eggs: 13 g/d Fish & seafood: 28 g/d Plant oils: 40 g/d Starchy vegetables: 50 g/d	Reaches recommended intake: **2 points** Exceeds recommended range but < 1x OR cosumes lower than recommended intake: **1 point** No intake or exceeds by ≥ 1x: **0 points**
**Red Light Foods** (Limited)	Red meat, animal oils, added sugars	Red meat: ≤ 14 g/d Animal oils: ≤ 11.8 g/d Added sugars: ≤ 31 g/d	≤ Recommended amount: **2 points** Exceeds recommended amount but < 1x: **1 point** Exceeds by ≥ 1x: **0 points**

Whole grains, in particular, take precedence over refined grains due to their superior nutritional content, including higher fibre and micronutrient levels, which contribute to both satiety and metabolic health.^(24–26)^ Fish and seafood, while included as a protein source, are recommended in moderation.^(27,28)^ This aligns with sustainability goals to limit overfishing while providing essential omega-3 fatty acids.^(27,29)^ In contrast, red and processed meat consumption is minimised or eliminated due to their high environmental impact, including greenhouse gas emissions and resource-intensive production. Plant-based protein sources, such as legumes and nuts, serve as sustainable alternatives.

Reducing the intake of added sugars and ultra-processed foods is another critical aspect of the Sustainable Plate. These foods contribute disproportionately to health issues such as obesity and type 2 diabetes,^(30)^ while their production often carries significant environmental costs.^(31)^ Minimising these items not only aligns with nutritional goals but also reduces the environmental footprint of diets. However, industrial food processing plays a key role in global food security, and the focus should be on reformulating processed foods to improve their nutritional quality rather than eliminating them. Developing affordable, nutrient-dense, and sustainable alternatives can help maintain the convenience of processed foods while promoting healthier dietary patterns.

The Sustainable Plate also emphasises local and seasonal food choices, which reduce transportation emissions and support local food systems. Incorporating regionally grown produce ensures dietary patterns are culturally adaptable while fostering community resilience. Furthermore, consideration of the environmental impact of food choices, such as greenhouse gas emissions, water usage, and land use, is integral to the framework, encouraging the selection of foods with lower ecological footprints. Finally, addressing food waste is an essential component of a Sustainable Plate. A report by the United Nations Environment Programme (UNEP) highlights that approximately 19% of global food production, equating to 1.05 billion metric tonnes, was wasted in 2022.^(32)^ This waste contributes significantly to greenhouse gas emissions and exacerbates food insecurity, with 783 million people facing chronic hunger.^(32)^ Therefore, reducing waste at all levels, from production to individual consumption, mitigates the inefficiencies of food systems and supports long-term sustainability.

**Fig. 1.**
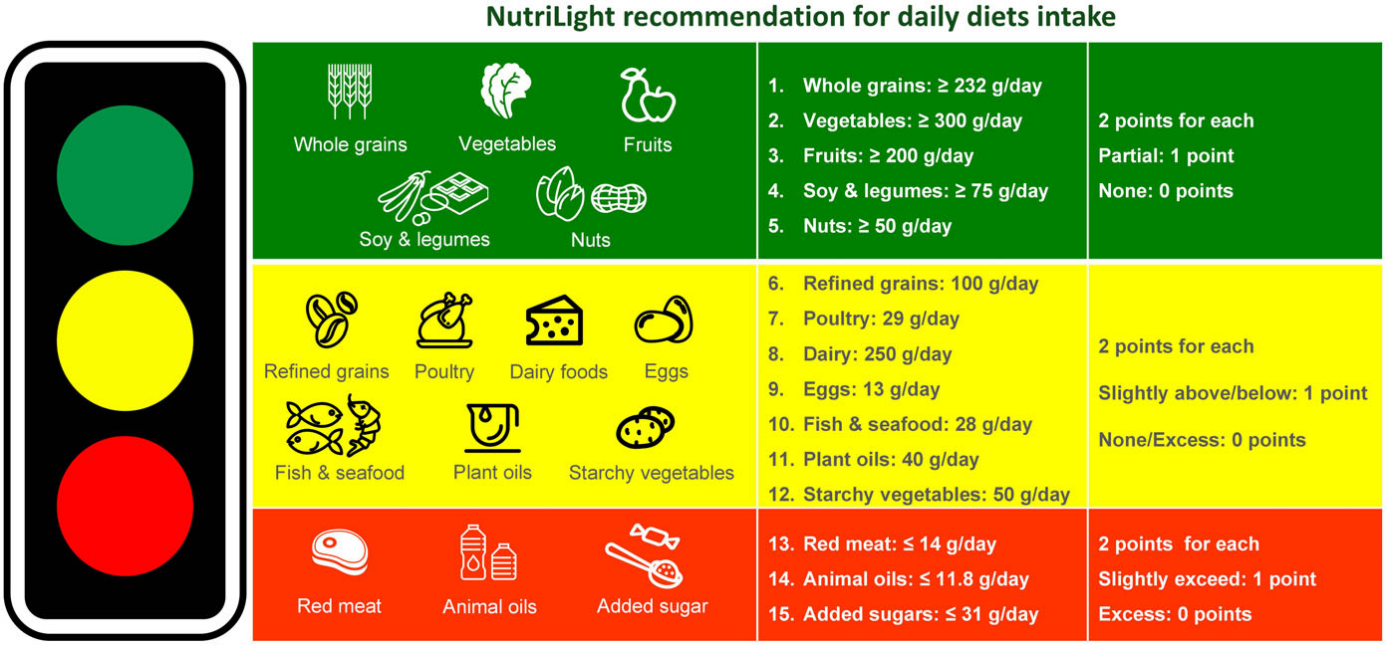
NutriLight recommendations: a traffic light framework for balanced and sustainable daily diets. *Note*: Points for each food group in Fig. 1 are calculated based on the scoring criteria outlined in Table 2. Green-light foods receive 2 points when meeting the recommended intake, 1 point for partial intake (0 < X < R), and 0 points if absent. Yellow-light foods receive 2 points for meeting the recommendation, 1 point for slightly above/below intake (0 < X < R or R < X < 2R), and 0 points if under- or overconsumed. Red-light foods receive 2 points for staying within the recommended limit, 1 point for slightly exceeding (R < X < 2R), and 0 points for excess consumption.

Effectively communicating the principles of the Sustainable Plate is critical for its widespread adoption. Simplified tools such as visual guides, including the traffic light categorisation system, make recommendations accessible and actionable for diverse audiences (Fig. 1). Behavioural nudges, such as default options in cafeterias that prioritise green light foods or incentives for sustainable purchases, can subtly guide individuals toward healthier and more sustainable choices.^(32–34)^ Additionally, using digital platforms to disseminate these tools can expand their reach, particularly in populations with varying levels of health literacy.^(35,36)^ Tailoring messages to specific cultural and regional contexts ensures that sustainable dietary practices are both understood and embraced, ultimately fostering a collective movement toward health and sustainability.

## Methodological considerations

The NutriLight recommendation, which classifies foods into green, yellow, and red categories with specific scoring criteria, offers a structured approach for dietary guidance (Fig. 1). To effectively implement and assess this system, suitable methodologies must be tailored to its unique characteristics.

Quantifying sustainability in diets, particularly within the NutriLight framework, presents several challenges. One primary obstacle is the complexity of dietary patterns, as food choices involve interactions across multiple food groups and nutrients. Balancing simplicity and accuracy in scoring systems is critical to ensuring their usability without oversimplifying the nuanced relationship between diet and sustainability.^(37–39)^ To address this, further validation analyses are warranted to assess the accuracy and relevance of the NutriLight scoring system.^(39)^ These could include comparisons with established dietary indices, such as the Healthy Eating Index (HEI) and Alternative Healthy Eating Index (AHEI), to evaluate alignment with recognised dietary quality measures. Additionally, inter-rater and intra-rater reliability testing should be conducted to ensure scoring consistency, while longitudinal studies should examine associations between NutriLight scores and health markers, including body mass index (BMI), metabolic profiles, and disease risks. Scores can be aggregated over time (i.e. by averaging scores, analysing trends in adherence, or classifying based on cumulative scores over a given period) to provide a more comprehensive evaluation of long-term dietary quality.

The challenges of assessing the NutriLight system are analogous to those encountered in dietary diversity evaluations.^(40)^ Both require multidimensional approaches to capture the balance, variety, and adequacy of food choices.^(40,41)^ For instance, metrics of dietary diversity, which consider the count, evenness, and dissimilarity of consumed foods, parallel the NutriLight system’s emphasis on categorising foods based on nutritional adequacy and environmental impact. Drawing from methods in dietary diversity research, NutriLight scoring could benefit from incorporating metrics that reflect the proportional balance of green, yellow, and red food categories, ensuring a holistic assessment of dietary sustainability. While the NutriLight system allows for dietary flexibility, it incorporates scoring mechanisms to encourage balanced intake across food groups, reducing the risk of unstructured, unhealthy choices. Details of the NutriLight scoring methods are shown in the Table 2a and 2b.

**Table 2.**
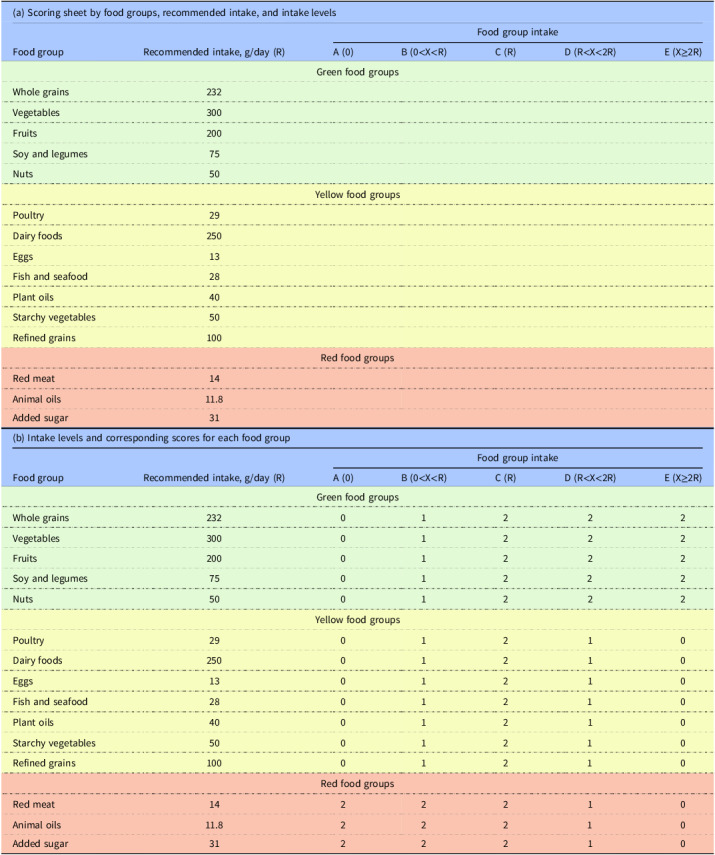
NutriLight scoring framework and scoring methods

*Note*: Scoring rule: Each food group is scored 0-1-2, with a total score of 0–30 across 15 food groups.

## Applications in public health

The Sustainable Plate provides a practical and scalable framework that can be integrated into public health recommendations and national dietary guidelines to address pressing health and environmental challenges. Incorporating the Sustainable Plate into public health recommendations can transform the way dietary guidelines are designed and communicated. For example, visual tools like the traffic light system can be used in public health campaigns, schools, and food labelling to encourage healthier food choices. Policymakers can also leverage the Sustainable Plate framework to shape food systems, such as by incentivizing the production and consumption of green-light foods like fruits, vegetables, legumes, and whole grains through subsidies and tax benefits. Additionally, embedding the Sustainable Plate principles into national dietary guidelines ensures consistency in health communication while promoting sustainable food systems, aligning individual choices with broader planetary health goals.

The implications for vulnerable populations and global food security are significant. Vulnerable groups, such as low-income households, children, and older adults, often face barriers to accessing nutritious and affordable foods. The Sustainable Plate framework advocates the importance of making green light foods more accessible and affordable through targeted interventions, such as government subsidies or community-based programmes. For example, policies that reduce the cost of nutrient-dense, plant-based foods can address disparities in diet quality and health outcomes among disadvantaged populations. Furthermore, the Sustainable Plate can guide food assistance programmes by prioritising the inclusion of green-light foods to improve both nutritional adequacy and long-term health outcomes.

## Future research directions

Current literature underscores the complexity of aligning dietary health benefits with environmental sustainability. While plant-based diets are generally associated with positive health outcomes and reduced environmental impacts, certain trade-offs exist.^(42,43)^ For example, some studies suggest that higher diet quality, as measured by indices like the HEI and AHEI, may be associated with increased total food demand and food loss and waste, potentially offsetting environmental gains.^(44)^ Further research is needed to delineate these trade-offs and develop dietary recommendations that optimise both health and sustainability outcomes.

The effectiveness of dietary interventions is significantly influenced by cultural, socioeconomic, and geographic factors. Existing dietary patterns, such as the Mediterranean or DASH diets, often reflect Western norms and may not align with the food traditions or preferences of other regions. This cultural mismatch can lead to resistance or misperceptions about the relevance of such diets. Additionally, language barriers and a lack of localised educational materials make it difficult to reach diverse populations, while social norms, such as reliance on animal-based diets, can conflict with the principles of plant-based patterns like veganism. Further studies should explore how simplified visual dietary tools, such as traffic-light approach, influence consumer food choices in everyday settings. Additionally, research is needed to assess the effectiveness of culturally adapted versions of the Sustainable Plate in different populations, ensuring that the framework remains practical and relevant in diverse food environments.

To robustly assess the impact of adopting the Sustainable Plate framework, longitudinal studies are warranted to evaluate how self-selected diets within the NutriLight system impact nutritional adequacy and dietary balance, reducing the risk of unhealthy choices. These studies should monitor participants over extended periods to evaluate changes in health outcomes, such as reductions in chronic disease incidence, improvements in nutritional status, and weight management. Simultaneously, environmental metrics, including carbon footprint, water usage, and biodiversity impact, should be measured to determine the ecological benefits of sustained dietary changes. Large-scale dietary surveys and real-world monitoring of NutriLight adoption through food consumption patterns, nutritional assessments, and purchasing data could provide valuable insights into its long-term feasibility and impact. Such comprehensive studies will provide empirical evidence on the long-term viability and effectiveness of the Sustainable Plate in promoting health and environmental sustainability. In addition, in the practical application of NutriLight system, some visual pictures should be included to help participants understand the portion sizes and make the right choices. By addressing these research areas, future studies can enhance the implementation of the Sustainable Plate framework, ensuring it effectively balances health and sustainability while being adaptable to the diverse needs of global populations.

In conclusion, the Sustainable Plate framework offers a practical and evidence-based approach to align human health and environmental sustainability. By prioritising plant-based, nutrient-dense foods while moderating resource-intensive and less health-promoting options, it provides actionable guidance for individuals and policymakers. Its adaptability across cultural contexts and integration into public health recommendations positions it as a critical tool for addressing diet-related diseases and ecological degradation. Future research should focus on resolving trade-offs between health and sustainability, tailoring interventions to diverse populations, and conducting longitudinal studies to evaluate its long-term impact on health and environmental metrics. The Sustainable Plate has the potential to transform global dietary practices, fostering a more resilient and equitable food system while safeguarding planetary health.
